# Assessment of Latent Subgroups With Suicidal Ideation and Suicidal Behavior Among Gun Owners and Non–Gun Owners in the US

**DOI:** 10.1001/jamanetworkopen.2022.11510

**Published:** 2022-05-11

**Authors:** Craig J. Bryan, AnnaBelle O. Bryan, Heather M. Wastler, Lauren R. Khazem, Ennio Ammendola, Justin C. Baker, Edwin Szeto, Jeffrey Tabares, Christina R. Bauder

**Affiliations:** 1 Department of Psychiatry and Behavioral Health, The Ohio State University College of Medicine, Columbus

## Abstract

**Importance:**

Firearm ownership is associated with increased risk for suicide.

**Objective:**

To examine patterns of associations among suicidal thoughts and behaviors among gun owners and non–gun owners in the US.

**Design, Setting, and Participants:**

In this survey study, cross-sectional online survey data were collected from March to April 2020 from US adults recruited via Qualtrics Panels. Quota sampling was used to approximate US census demographics.

**Main Outcomes and Measures:**

The primary outcomes were past-year passive suicidal ideation, active suicidal ideation, suicidal planning, suicidal behaviors, and nonsuicidal self-injury as measured by items from the Self-Injurious Thoughts and Behaviors Interview–Revised (SITBI-R). Simple latent class analysis (LCA) was used to assign participants to separate classes based on posterior probabilities, and multigroup LCA was used to assess whether the same construct was measured in specified groups.

**Results:**

Of 65 079 adults invited to participate, 10 625 (16.3%) completed the survey; 9153 responded “yes” or “no” to the firearm ownership item and were included in the analysis. Of these 9153 respondents (4695 [51.3%] male; mean [SD] age, 46.7 [16.8] years), 2773 (30.3%) reported owning a gun and 6380 (69.7%) reported not owning a gun. Compared with non–gun owners, gun owners were more likely to be male (1779 [64.2%] vs 2916 [45.7%]; χ^2^_1_, 263.3; *P* < .001) and White (2090 [75.4%] vs 3945 [61.8%]; χ^2^_5_, 232.9; *P* < .001) and to have served in the military (772 [27.8%] vs 609 [9.5%]; χ^2^_1_, 571.4; *P* < .001). Five distinct patterns of SITBI-R item endorsement were extracted using simple LCA. Multigroup LCA indicated that the probability of SITBI-R item endorsement differed between gun owners and non–gun owners across subgroups. Among gun owners, the probability of past-month nonfatal suicide attempts was highest in class 4 (ranging from 16.8% for reaching out for help to 27.2% for starting, then changing one’s mind). Gun owners in class 4 were characterized by high probabilities of endorsing thoughts about specific ways or methods to attempt suicide (100%) and preparatory behavior (100%). Among non–gun owners, the probability of nonfatal suicide attempts was highest in class 5 (ranging from 14.9% for reaching out for help to 29.7% for starting, then changing one’s mind). Non–gun owners in class 5 were characterized by high probabilities of endorsing passive suicidal ideation (84.0%-100%), active suicidal ideation (86.7%-95.0%), and thoughts about specific ways or methods to attempt suicide (97.4%) and a specific place (92.1%) to attempt suicide.

**Conclusions and Relevance:**

In this study, in subgroups with lower probabilities of suicide attempt, gun owners and non–gun owners showed similar patterns of suicide risk item endorsement, but when the probability of a suicide attempt increased, gun owners were less likely than non–gun owners to endorse passive and active suicidal ideation. These findings suggest that assessing a broader range of suicide risk indicators may improve risk detection.

## Key Points

**Question:**

Do survey item response patterns for suicidal ideation and behaviors differ between adult gun owners and non–gun owners in the US?

**Findings:**

This survey study of 10 625 US adults identified 5 response patterns of passive and active suicidal ideation, suicidal planning, and suicidal behavior. Response patterns were similar among gun owners and non–gun owners in subgroups with lower probabilities of suicidal behavior but differed in subgroups with higher probabilities of suicidal behavior.

**Meaning:**

The findings suggest that gun owners with elevated risk of suicidal behavior are less likely to be detected by screening tools that ask about suicidal ideation.

## Teaser

This survey study investigates whether patterns of reported suicidal ideation, planning, and behavior differ among US adults who own a firearm vs those who do not own a firearm.

## Introduction

The US suicide rate has increased by more than 30% since 2000,^1^ with firearms being the leading method of suicide.^2^ Although nonfatal suicidal behaviors and suicidal ideation are correlated with suicide mortality,^3^ the varied ways in which suicidal ideation and suicidal behavior co-occur remain poorly understood. For example, many people who attempt suicide deny experiencing suicidal ideation and suicidal planning in advance,^4,5,6,7,8,9^ in some cases because the language used in screening tools does not reflect their subjective experience of suicidal thoughts.^10^ Additional research focused on examining heterogeneity in patterns of suicidal ideation and suicidal behavior is therefore warranted.

In the US, gun ownership is associated with elevated suicide rates,^11,12,13^ in part because suicidal behavior involving firearms is more likely to be fatal.^14^ Suicidal ideation and suicidal behaviors may therefore be more likely to result in death among gun owners than among non–gun owners. Some studies suggest that gun owners are more likely to endorse suicidal ideation and nonfatal suicide attempts than are non–gun owners,^15,16^ but other studies suggest no differences in endorsement rates.^11,16,17^ These inconsistencies may be attributable to the use of single-item assessment measures that are especially vulnerable to measurement error^9^; even minor language changes in item wording, for example, have been shown to influence self-disclosure of suicidal ideation.^9,18,19^ Use of multiple items to assess suicidal ideation and behaviors may therefore improve construct measurement.

This study aimed to address these gaps by assessing a broad range of suicidal ideation and behaviors among US adults who own and do not own firearms. We hypothesized that with use of multigroup latent class analysis (LCA), multiple configurations of suicidal ideation, suicidal planning, and nonfatal suicidal behavior could be empirically identified and that these configurations would differ between gun owners and non–gun owners.

## Methods

### Participants and Procedures

This survey study followed the American Association for Public Opinion Research (AAPOR) reporting guideline. The study was approved by the institutional review board of the University of Utah. Participants included US adults recruited via Qualtrics Panels,^20^ an online survey platform that maintains a database of millions of US residents. Quota sampling was used to recruit a sample that was demographically similar (within 10%) to 2010 US census data percentages for age, sex, race and ethnicity, income, and educational level. Inclusion criteria were (1) age 18 years or older, (2) residency in the US, and (3) ability to speak and understand English. No exclusion criteria were used. Panel members first reviewed an information page describing the study’s purpose (ie, “to examine how the kinds of stress that adults experience in their lives can influence how they make decisions”), potential risks and benefits of participation, and investigator contact information. After reading the information page, panel members selected a radio button designating their consent to participate and allowing them to begin the survey. Participants who completed the full survey received compensation in the amount they agreed to when signing up for the survey panel (eg, cash, airline miles, or gift cards). Data were collected from March to April 2020.

Because data were collected online, multiple data quality procedures were used, including attention and effort checks (ie, examination of straight-line responding on scales with reverse-scored items and of inconsistent response patterns), a restriction of only 1 response per internet provider address, and use of CAPTCHA (Completely Automated Public Turing test to tell Computers and Humans Apart) images to reduce bot-generated responses. In addition, email invitations were sent to potential participants in batches so that data quality could be continuously reviewed.

### Measures

#### Self-Injurious Thoughts and Behaviors Interview–Revised

The Self-Injurious Thoughts and Behaviors Interview–Revised (SITBI-R)^21^ uses multiple items to assess passive suicidal ideation (thoughts about death or wishing to not exist), active suicidal ideation (thoughts about engaging in suicidal behavior), suicidal planning (thinking about the specific method, time, and location of suicidal behavior), nonsuicidal self-injury (NSSI) (intentional self-directed violence without any expectation or intent to die as a result), and suicidal behaviors (intentional self-directed violence with some expectation or intent to die as a result). Item content is shown in the Box. Although NSSI is not a form of suicidal behavior, we included this variable to examine whether this behavior co-occurred with suicidal thoughts and behaviors.

Box. **Self-Injurious Thoughts and Behaviors Interview–Revised Items Used to Assess Suicidal Ideation and Suicidal Behavior^a^**

**Passive Suicidal Ideation**

1. Have you ever had any of the following thoughts for more than a few minutes? Choose all that you have experienced:
  - I wish I could disappear or not exist.
  - I wish I was never born.
  - My life is not worth living.
  - I wish I could go to sleep and never wake up.
  - I wish I were dead.

**Active Suicidal Ideation**

1. Have you ever had thoughts of killing yourself?
2. Have you ever had any of the following thoughts for more than a few minutes? Choose all that you have experienced:
  - Maybe I should kill myself.
  - I should kill myself.
  - I am going to kill myself.

**Suicidal Planning**

1. Have you ever thought of any of the following?
  - A specific way or method to kill yourself.
  - A specific place to kill yourself.
  - A specific time to kill yourself.

**Suicidal Behavior**

1. Have you ever made an actual attempt to kill yourself in which you had at least some intent to die?
2. Have you ever done any of the following? Please read each item carefully. Some items describe behaviors that are similar to each other, but each item is asking about something different. We have provided some examples of each to help you understand each item. Choose all that you have done:
  - Done something to prepare to make a suicide attempt. For example, gaining access to method, looking up a method online, writing a suicide note, updating your will, or saying goodbye to loved ones.
  - Practiced making a suicide attempt. For example, taking an overdose of an amount you knew in advance would not kill you, hanging a rope to see if it would support your body weight, or jumping from a height that you knew in advance would not kill you.
  - Been very close to killing yourself but at the last minute you decided not to do it before taking any action. For example, holding a bottle of pills in your hand but deciding not to take any, setting up a noose but then deciding not to use it, or pointing a gun to your head.
  - Started to kill yourself and then you stopped after you had already taken some action. For example, taking 1 or a few pills of an overdose but then you changed your mind and stopped yourself or starting to hang yourself but then you changed your mind and found a way down.
  - Started to kill yourself and then you decided to reach out for help after you had already taken some action. For example, taking an overdose and then calling a friend or 911 for help.
  - Tried to kill yourself and someone found you afterward. For example, taking an overdose of pills and then someone found you unconscious and called 911 or hanging yourself and then someone found you and cut you down.
  - Tried to kill yourself and no one found you afterward. For example, taking an overdose of pills and then waking up later.

**Nonsuicidal Self-injury**

1. Have you ever done any of the following? Please read each item carefully. Some items describe behaviors that are similar to each other, but each item is asking about something different. We have provided some examples of each to help you understand each item. Choose all that you have done:
  - Purposely hurt yourself without wanting to die. For example, cutting yourself or burning your skin to reduce emotional stress, hitting yourself on purpose, punching a wall, or picking a fight with someone so you could feel physical pain.

^a^
Questions and response options are from the Self-Injurious Thoughts and Behaviors Interview–Revised.^21^ Response options for each item were “yes” or “no,” as in the original source. Participants selecting “yes” for any item were then asked when they most recently experienced each thought or behavior (within the past month, within the past year, or >1 year ago); the time frame responses were revised for this study.

Self-report versions of the SITBI-R have demonstrated good reliability and validity compared with an interview version.^21^ Participants were first asked to report whether they had experienced each item listed in the Box at any point in their lives (yes or no). All items were administered to all participants regardless of their answers to previous items. Participants selecting “yes” for an item were then asked when they had most recently experienced the item (within the past month, within the past year, or >1 year ago). Responses were dichotomized as more than 1 year ago (0) vs within the past month or within the past year (1). Past-year occurrence was selected to balance recency (to reduce recall bias) with statistical power for the planned analyses.

#### Demographics and Firearm Ownership

Multiple self-reported demographic variables were assessed: sex, gender, race and ethnicity, Hispanic/Latino ethnicity, history of military service, highest educational level, annual household income, and age. Firearm ownership was assessed with the following self-report item: “Do you personally own a gun or firearm?” Response options were “yes,” “no,” “don’t know/unsure,” and “refuse to answer.” Participants selecting “yes” were classified as gun owners, whereas participants selecting “no” were classified as non–gun owners. Participants selecting “don’t know/unsure” or “refuse to answer” were excluded from analyses.

### Statistical Analysis

All analyses were conducted using MPlus software, version 8.3 (Muthén & Muthén).^22^ Binary SITBI-R item responses were entered as variables. We first used simple LCA with a robust maximum likelihood estimator to assign participants to separate classes based on posterior probabilities. We estimated a series of models with 2 to 5 classes using robust maximum likelihood and selected the optimal number of classes based on the following criteria: (1) a minimum entropy value of 0.80, with values closer to 1.00 indicating better differentiation of classes^23,24^; (2) a comparison of bayesian information criterion values, with smaller values indicating better fit^25,26^; (3) the Lo-Mendell-Rubin likelihood ratio test to compare models with *k* and *k +* 1 classes,^27^ with a statistically significant test suggesting the model with *k +* 1 classes was better and a nonsignificant test suggesting the model with *k* classes was better; and (4) the correct-model-probability statistic, which provides estimates of each model being correct among all models considered, with higher values indicating greater probability of the model being correct.^28^ After the optimal model was identified, we used multigroup LCA, which allows class membership and item response probabilities to differ across groups, thereby providing a method for assessing whether the same construct was being measured in specified groups. Self-reported firearm ownership (yes or no) was selected as the grouping variable. Complete data (ie, SITBI-R item responses) were available from all participants. All significance tests were interpreted using 2-tailed *P* < .05.

## Results

Of 65 079 panel members invited to participate, 10 625 completed the survey (16.3% participation rate). A total of 69 respondents (0.6%) selected “don’t know/unsure” and 1403 (13.2%) declined to answer or skipped the item and were excluded from the analysis. Subsequent analyses included the 9153 participants (4695 [51.3%] male; mean [SD] age, 46.7 [16.8] years) who responded either “yes” or “no” to the firearm ownership item; 6380 (69.7%) reported not owning a gun, and 2773 (30.3%) reported owning a gun. Demographic characteristics are shown in Table 1 and were within the expected margin of error for US census estimates. Gun owners were more likely than non–gun owners to identify as male (1779 [64.2%] vs 2916 [45.7%]; χ^2^_1_, 263.3; *P* < .001), White (2090 [75.4%] vs 3945 [61.8%]; χ^2^_5_, 232.9; *P* < .001), and non-Hispanic/Latino (2104 [75.9%] vs 4526 [70.9%]; χ^2^_1_, 23.6; *P* < .001) and were more likely to have served in the military (772 [27.8%] vs 609 [9.5%]; χ^2^_1_, 571.4; *P* < .001). Gun owners were also slightly older (mean [SD] age, 47.4 [15.5] years vs 46.5 [17.3] years; *F*_1,9147_, 6.4; *P* = .01).

**Table 1.** Characteristics of the Sample Overall and by Gun Ownership

Variable	Participants, No. (%)			Gun owners vs non–gun owners		
	Total (N = 9153)	Gun owners (n = 2773)	Non–gun owners (n = 6380)	χ^2^ or *F*^a^	*P* value	Φ or η^2^^b^
Age, mean (SD), y	46.7 (16.8)	47.4 (15.5)	46.5 (17.3)	6.4	.01	0.001
Sex						
Female	4458 (48.7)	994 (35.8)	3464 (54.3)	263.3	<.001	0.17
Male	4695 (51.3)	1779 (64.2)	2916 (45.7)			
Gender						
Female	4435 (48.5)	991 (35.7)	3444 (54.0)	262.0	<.001	0.17
Male	4677 (51.1)	1771 (63.9)	2906 (45.5)			
Transgender	26 (0.3)	9 (0.3)	17 (0.3)			
Other	15 (0.2)	2 (0.1)	13 (0.2)			
Race and ethnicity						
Asian	658 (7.2)	91 (3.3)	567 (8.9)	232.9	<.001	0.16
Black	1062 (11.6)	312 (11.3)	750 (11.8)			
Native American	316 (3.5)	92 (3.3)	224 (3.5)			
Pacific Islander	70 (0.8)	23 (0.8)	47 (0.7)			
White	6035 (65.9)	2090 (75.4)	3945 (61.8)			
Other	1012 (11.1)	165 (6.0)	847 (13.3)			
Hispanic/Latino ethnicity						
No	6630 (72.4)	2104 (75.9)	4526 (70.9)	23.6	<.001	−0.05
Yes	2523 (27.6)	669 (24.1)	1854 (29.1)			
Military service						
No	7772 (84.9)	2001 (72.2)	5771 (90.5)	571.4	<.001	0.25
Yes						
In the past	1167 (12.7)	602 (21.7)	565 (8.9)			
Current	214 (2.3)	170 (6.1)	44 (0.7)			
Educational level						
Some high school	316 (3.5)	47 (1.7)	269 (4.2)	95.0	<.001	0.10
GED	228 (2.5)	56 (2.0)	172 (2.7)			
High school diploma	1621 (17.7)	414 (14.9)	1207 (18.9)			
Degree						
Associate’s	1193 (13.0)	431 (15.5)	762 (11.9)			
Bachelor’s	2222 (24.3)	707 (25.5)	1515 (23.7)			
Graduate	1468 (16.0)	512 (18.5)	956 (15.0)			
Annual income, $						
<25 000	1536 (16.8)	213 (7.7)	1323 (20.7)	385.7	<.001	0.21
25 000-34 999	876 (9.6)	196 (7.1)	680 (10.7)			
35 000-49 999	908 (9.9)	244 (8.8)	664 (10.4)			
50 000-74 999	1823 (19.9)	549 (19.8)	1274 (20.0)			
75 000-99 999	1303 (14.2)	480 (17.3)	823 (12.9)			
100 000-149 999	1582 (17.3)	617 (22.3)	965 (15.1)			
150 000-199 999	614 (6.7)	264 (9.5)	350 (5.5)			
≥200 000	511 (5.6)	210 (2.3)	301 (3.3)			

Abbreviation: GED, General Educational Development.

^a^ For categorical variables (rates or percentages), χ^2^ statistics are given, and for continuous variables (means), *F* statistics are given.

^b^ For categorical variables (rates or percentages), Φ statistics are given, and for continuous variables (means), η^2^ statistics are given.

### Rates of Past-Year Self-injurious Thoughts and Behaviors

Gun owners were more likely than non–gun owners to endorse 2 of 5 passive suicidal ideation items, 3 of 4 active suicidal ideation items, 3 of 3 suicidal planning items, 9 of 9 suicidal behavior items, and the NSSI item (Table 2). The largest differences were for preparatory behavior (odds ratio [OR], 3.75; 95% CI, 3.06-4.59), attempting to kill oneself with some intent to die (OR, 3.75; 95% CI, 2.95-4.76), practice or rehearsal behavior (OR, 3.42; 95% CI, 2.60-4.50), and making a suicide attempt but being found afterward (OR, 3.57; 95% CI, 2.34-5.44).

**Table 2.** Rates of Self-Injurious Thoughts and Behaviors Interview–Revised Item Endorsement Overall and by Gun Ownership Status

Item	Participants, No. (%)			OR (95% CI)	*P* value
	Total (N = 9153)	Gun owners (n = 2773)	Non–gun owners (n = 6380)		
Passive suicidal ideation					
I wish I could disappear or not exist	1054 (11.5)	341 (12.3)	713 (11.2)	1.11 (0.97-1.28)	.12
I wish I was never born	581 (6.3)	212 (7.6)	369 (5.8)	1.35 (1.13-1.61)	.001
My life is not worth living	651 (7.1)	245 (8.8)	406 (6.4)	1.43 (1.21-1.68)	<.001
I wish I could go to sleep and never wake up	929 (10.1)	299 (10.8)	630 (9.9)	1.10 (0.95-1.28)	.19
I wish I were dead	537 (5.9)	178 (6.4)	359 (5.6)	1.15 (0.96-1.39)	.14
Active suicidal ideation					
Thoughts of killing yourself	918 (10.0)	352 (12.7)	566 (8.9)	1.49 (1.30-1.72)	<.001
Maybe I should kill myself	439 (4.8)	165 (6.0)	274 (4.3)	1.41 (1.16-1.72)	.001
I should kill myself	294 (3.2)	120 (4.3)	174 (2.7)	1.61 (1.27-2.05)	<.001
I am going to kill myself	191 (2.1)	66 (2.4)	125 (2.0)	1.22 (0.90-1.65)	.20
Suicidal planning					
Specific way or method	647 (7.1)	295 (10.6)	352 (5.5)	2.04 (1.73-2.40)	<.001
Specific place	340 (3.7)	154 (5.6)	186 (2.9)	1.96 (1.57-2.44)	<.001
Specific time	220 (2.4)	97 (3.5)	123 (1.9)	1.84 (1.41-2.42)	<.001
Suicidal behaviors					
Attempt to kill self with some intent to die	291 (3.2)	177 (6.4)	114 (1.8)	3.75 (2.95-4.76)	<.001
Preparatory behavior	411 (4.5)	248 (8.9)	163 (2.6)	3.75 (3.06-4.59)	<.001
Practice or rehearsal behavior	217 (2.4)	128 (4.6)	89 (1.4)	3.42 (2.60-4.50)	<.001
Aborted attempt	227 (2.5)	114 (4.1)	113 (1.8)	2.74 (1.83-3.10)	<.001
Interrupted attempt	145 (1.6)	71 (2.6)	74 (1.2)	2.24 (1.61-3.11)	<.001
Suicide attempt					
Started, then changed mind	115 (1.3)	55 (2.0)	60 (0.9)	2.13 (1.47-3.08)	<.001
Started, then reached out for help	82 (0.9)	41 (1.5)	41 (0.6)	2.32 (1.50-3.59)	<.001
Found afterward	91 (1.0)	55 (2.0)	36 (0.6)	3.57 (2.34-5.44)	<.001
Not found afterward	83 (0.9)	37 (1.3)	46 (0.7)	1.86 (1.21-2.88)	.004
Nonsuicidal self-injury					
Hurting self without wanting to die	307 (3.4)	116 (4.2)	191 (3.0)	1.42 (1.12-1.79)	.004

Abbreviation: OR, odds ratio.

### Latent Class Analysis Results

Results of the simple LCA supported a 5-class model (eTables 1 and 2 in the Supplement). Results of the multigroup LCA are summarized in Table 3. Among gun owners, class 1 was well characterized by an absence of passive suicidal ideation, active suicidal ideation, suicidal planning, suicidal behavior, and NSSI. Class 2 was well characterized by an absence of suicidal behavior and modestly characterized by the wish to disappear or not exist and to go to sleep and never wake up. Class 3 was not well characterized by any item or construct. Class 4 was well characterized by an absence of the wish to go to sleep and never wake up and the presence of thoughts about specific ways or methods to attempt suicide and preparatory behavior. Class 4 was also modestly characterized by the wish to disappear or not exist, active thoughts of killing oneself, and attempts to kill oneself with some intent to die. Among gun owners, respondents in class 4 had the greatest probability of recently attempting suicide; 16.8% to 63.3% of this subgroup endorsed a suicide attempt item. The probability of past-month nonfatal suicide attempts was highest in class 4 (ranging from 16.8% for reaching out for help to 27.2% for starting an attempt, then changing one’s mind). Gun owners in class 4 were characterized by high probabilities of endorsing thoughts about specific ways or methods to attempt suicide (100%) and preparatory behavior (100%). Class 5 was well characterized by all but 1 passive ideation item (ie, I wish I was never born), active thoughts of killing oneself and ambivalent thoughts of killing oneself, and thinking about specific ways or methods to attempt suicide.

**Table 3.** Self-Injurious Thoughts and Behaviors Interview–Revised Item Response Probabilities Across 5 Latent Classes by Gun Ownership Status

Item	Class, %^a^				
	1	2	3	4	5
**Gun owners**					
Total	2360	115	231	30	37
Passive suicidal ideation					
I wish I could disappear or not exist	1.0	62.4	21.2	63.3	84.4
I wish I was never born	0.2	26.0	17.3	23.6	59.2
My life is not worth living	0.7	41.1	9.7	33.5	78.5
I wish I could go to sleep and never wake up	0.9	63.9	14.9	0	100
I wish I were dead	0.1	28.7	9.9	22.1	88.4
Active suicidal ideation					
Thoughts of killing yourself	0.4	45.8	22.2	61.1	89.7
Maybe I should kill myself	0	20.6	6.6	22.8	89.2
I should kill myself	0	10.1	9.7	22.7	71.3
I am going to kill myself	0	2.8	1.3	20.1	50.4
Suicidal planning					
Specific way or method	0.1	22.4	23.4	100	83.2
Specific place	0	11.2	10.6	10.1	63.8
Specific time	0.1	10.0	6.7	20.0	44.8
Suicidal behavior					
Attempt to kill self with some intent to die	0.1	0.8	19.7	63.3	27.3
Preparatory behavior	0.3	3.1	42.0	100	55.8
Practice or rehearsal behavior	0.3	0	10.2	13.6	16.3
Aborted attempt	0	0	15.5	16.1	15.8
Interrupted attempt	0.1	0	6.1	22.5	13.6
Suicide attempt					
Started, then changed mind	0.1	0	4.6	27.2	10.8
Started, then reached out for help	0	1.2	6.6	16.8	2.8
Found afterward	0.2	0	3.2	32.0	10.4
Not found afterward	0	0.9	1.9	29.0	7.7
Nonsuicidal self-injury					
Hurting self without wanting to die	0.5	11.3	5.9	20.0	16.4
**Non–gun owners**					
Total	5939	190	131	79	41
Passive suicidal ideation					
I wish I could disappear or not exist	1.2	69.4	24.4	85.2	89.3
I wish I was never born	0.3	29.1	16.6	56.2	84.0
My life is not worth living	0.3	31.1	14.9	71.4	86.2
I wish I could go to sleep and never wake up	0.9	60.0	14.0	78.0	97.0
I wish I were dead	0.2	22.3	13.5	83.7	100
Active suicidal ideation					
Thoughts of killing yourself	0.2	21.1	45.0	84.6	95.0
Maybe I should kill myself	0	5.5	31.1	67.1	87.6
I should kill myself	0	0	10.7	41.2	94.7
I am going to kill myself	0	2.1	4.0	19.6	86.7
Suicidal planning					
Specific way or method	0	4.9	39.9	60.7	97.4
Specific place	0	0.9	14.2	18.7	92.1
Specific time	0.1	0	10.9	9.6	73.8
Suicidal behavior					
Attempt to kill self with some intent to die	0.1	0.9	7.2	5.3	17.7
Preparatory behavior	0.1	0.9	26.0	7.0	52.2
Practice or rehearsal behavior	0	0	12.0	0	22.3
Aborted attempt	0	1.0	12.9	5.2	34.7
Interrupted attempt	0	0.2	9.5	5.8	27.5
Suicide attempt					
Started, then changed mind	0	0.4	6.7	0	29.7
Started, then reached out for help	0	0.8	2.3	0	14.9
Found afterward	0	0	4.1	0	14.8
Not found afterward	0	0	6.1	1.2	19.7
Nonsuicidal self-injury					
Hurting self without wanting to die	0.4	5.6	12.8	21.6	35.3

^a^ Simple latent class analysis with a robust maximum likelihood estimator was used to assign participants to separate classes based on posterior probabilities.

Among non–gun owners, class 1 was well characterized by the absence of passive suicidal ideation, active suicidal ideation, suicidal planning, suicidal behavior, and NSSI. Class 2 was well characterized by an absence of active suicidal ideation, suicidal planning, and suicidal behavior and modestly characterized by the wish to disappear or not exist and to go to sleep and never wake up. Class 3 was not well characterized by any item or construct. Class 4 was well characterized by passive suicidal ideation, active thoughts of killing oneself, and the absence of suicidal behaviors. Class 4 was also modestly characterized by ambivalent suicidal ideation and thoughts about specific ways or methods of attempting suicide. Class 5 was well characterized by passive suicidal ideation (84.0%-100%), active suicidal ideation (86.7%-95.0%), and thoughts about specific ways or methods to attempt suicide (97.4%) and a specific place (92.1%) to attempt suicide. Among non–gun owners, class 5 also had the greatest probability of endorsing a recent suicide attempt, ranging from 14.9% for reaching out for help to 29.7% for starting an attempt, then changing one’s mind.

## Discussion

Multiple studies have shown that firearm ownership and availability are positively correlated with suicide mortality at the population level.^12,13,29^ Less is known about how suicidal ideation, suicidal planning, and suicidal behavior co-occur within and among different subgroups, however. In this study, the overall probabilities of endorsing suicidal ideation, suicidal planning, and suicidal behavior were positively associated with one another, a pattern consistent with previous research.^3^ However, the specific patterns of co-occurrence varied considerably among latent classes and gun ownership groups. The probability of endorsing suicidal ideation, suicidal planning, and suicidal behavior therefore was associated in part with subgroup membership and gun ownership status, suggesting that gun owners and non–gun owners with similar levels of suicide risk were not necessarily equally likely to endorse survey items assessing a range of suicidal thoughts and behaviors.

Differences in item response probabilities may occur because item content or wording is interpreted differently by or functions in different ways among members of distinct subgroups.^30^ In this sample, for instance, gun owners in classes 3, 4, and 5 were more likely to endorse the suicide attempt item asking whether they had ever “made an actual attempt to kill yourself in which you had at least some intent to die” compared with the 4 suicide attempt items that included descriptions of a possible outcome of a suicide attempt (ie, being found or not found) and examples of each behavior (Box). Non–gun owners were likely to endorse these 2 types of suicide attempt items similarly. This suggests that the concept of suicide attempt may have different meanings for gun owners and non–gun owners and, furthermore, that gun owners’ responses may have been more susceptible to item language and content.

Although the gun owners in this study were generally more likely than non–gun owners to endorse passive and active suicidal ideation (a pattern consistent with previous research),^15^ this specific configuration was revealed only in the subgroups with lower probabilities of suicidal behavior (especially suicide attempts), including class 1, which was characterized by low endorsement probabilities of all items; class 2, which was characterized by low probability of suicidal behavior combined with moderate probability of passive suicidal ideation; and class 3, which was characterized by low probability of suicidal behavior combined with no clear pattern of passive suicidal ideation, active suicidal ideation, and suicidal planning. In subgroups with higher probabilities of suicidal behavior, however, item response patterns differed between gun owners and non–gun owners. Among gun owners, the highest probability of suicidal behavior occurred in the subgroup that was well characterized by thinking of specific ways or methods to attempt suicide and by preparatory behaviors but not by passive suicidal ideation or active suicidal ideation (class 4). Among non–gun owners the highest probability of suicidal behavior occurred in the subgroup that was well characterized by passive suicidal ideation, active suicidal ideation, and suicidal planning but not by preparatory behavior (class 5). Passive suicidal ideation, active suicidal ideation, and suicidal planning may therefore function similarly for gun owners and non–gun owners who have lower probabilities of suicidal behavior but may not be comparable when the probability of suicidal behavior is higher.

This finding has important implications for suicide risk screening methods, which typically include questions about thoughts of death (ie, passive ideation) and/or suicide (ie, active suicidal ideation).^31,32,33^ In subgroups with higher probabilities of suicide attempts, gun owners were less likely than non–gun owners to endorse passive and active suicidal ideation but were more likely to endorse thinking about specific ways or methods to attempt suicide and engaging in preparatory behaviors. This finding suggests that elevated risk states may be experienced by gun owners and non–gun owners in unique ways that differ sufficiently such that they cannot be meaningfully compared.

Of clinical importance, our results suggest that suicide risk screening and assessment methods that focus on self-disclosure of thoughts about death and suicide may be well suited for identifying high risk states among non–gun owners but that these screening items are less likely to be endorsed by gun owners with the highest probability of suicide attempt. Evidence has shown that suicidal ideation has only modest sensitivity as an indicator of suicidal behavior, even when someone is screened within a few weeks of their suicide attempt.^3,4,34,35,36^ The present study’s results suggest that the modest sensitivity of suicidal ideation as an indicator of suicidal behavior may be explained in part by heterogeneous associations among various indicators of suicide risk. The findings suggest that supplementing passive and active suicidal ideation items with items assessing thoughts about specific ways or methods of attempting suicide and/or taking steps to prepare for a suicide attempt may improve detection of subgroups at high risk of suicide attempt.

### Strengths and Limitations

Strengths of the present study include the large sample size and use of quota sampling, which allowed us to enroll enough participants with suicidal ideation, suicidal planning, and suicidal behavior to examine various combinations of these variables at the participant level. Although quota sampling does not necessarily produce samples that are representative of the population, the sample recruited in this study was within 10% of the US Census general population percentages^37^ for all demographic variables, and the estimated rate of firearm ownership in our study is very similar to the rate reported in a previous study using probability sampling.^11^

This study also has limitations. The cross-sectional design restricted our ability to examine directional associations among suicidal ideation, suicidal planning, and suicidal behaviors. For example, gun owners and non–gun owners may differ with respect to specific temporal patterns of emergence, such that passive suicidal ideation may have emerged before active suicidal ideation for some participants whereas active suicidal ideation may have emerged before passive suicidal ideation for others. We also were unable to make conclusions about causal relationships. Second, our survey assessed firearm ownership rather than firearm availability. *Firearm ownership* as a concept and term may be subject to a broader range of definitions and interpretations than *firearm availability*, which refers to physical proximity and/or access to a firearm. For example, a participant may live in a residence with someone who keeps a firearm, but the participant may not identify as a gun owner because they define *gun owner* as someone who acquires and then maintains personal responsibility for a firearm. A different participant with those same circumstances may instead identify as a gun owner because they define *gun owner* as someone who lives in a residence where a firearm is present. Further research is warranted to investigate whether the observed pattern of results is maintained when groupings are based on firearm availability or firearm ownership. Third, we were unable to ascertain whether firearms were obtained before or after the emergence of suicidal thoughts or behaviors. Fourth, because we did not ask participants to report the specific ways or methods of suicide they were considering, we were unable to determine whether specific ways or methods of suicide that were considered at higher rates among gun owners in some classes involved firearms or other possible suicide attempt methods.

## Conclusions

The results of this survey study may reveal new clues for understanding heterogeneity in suicide risk within and between populations of gun owners and non–gun owners. Our results suggest that configurations of suicidal ideation and behaviors are similar between gun owners and non–gun owners when the probability of suicide attempt is low, but when the probability of suicide attempt increases, gun owners may be less likely than non–gun owners to endorse survey items about passive and active suicidal ideation. The findings suggest that assessing a broader range of suicide risk indicators, especially thoughts about specific ways or methods to attempt suicide and preparation for a suicide attempt, may improve risk detection in subgroups at high risk of suicide attempt.

## Notes

Supplement. **eTable 1.** Fit Statistics for Latent Class Analysis Models **eTable 2.** SITBI-R Item Response Probabilities Across 5 Latent Classes
