# Supplementary material for: Assessment of Latent Subgroups With Suicidal Ideation and Suicidal Behavior Among Gun Owners and Non–Gun Owners in the US
Source: JAMA Netw Open. 2022 May 11;5(5):e2211510. doi: 10.1001/jamanetworkopen.2022.11510 (PMC9096594; doi:10.1001/jamanetworkopen.2022.11510)
Supplement: Supplement. — eTable 1. Fit Statistics for Latent Class Analysis Models eTable 2. SITBI-R Item Response Probabilities Across 5 Latent Classes [file jamanetwopen-e2211510-s001.pdf]

Supplementary Online Content

Bryan CJ, Bryan AO, Wastler HM, et al. Assessment of latent subgroups with suicidal ideation and suicidal behavior among gun owners and non–gun owners in the US. *JAMA Netw Open*. 2022;5(5):e2211510. doi:10.1001/jamanetworkopen.2022.11510

eTable 1. Fit Statistics for Latent Class Analysis Models
eTable 2. SITBI-R Item Response Probabilities Across 5 Latent Classes

This supplementary material has been provided by the authors to give readers additional information about their work.

© 2022 Bryan CJ et al. *JAMA Network Open*.

eTable 1. Fit statistics for latent class analysis models

No. of Classes	Entropy	BIC	Likelihood Ratio Test	cmP
2	0.970	38952.60	<.001	0
3	0.959	37820.93	<.001	1.22×10^{-88}
4	0.961	37471.34	<.001	9.96×10^{-13}
5	0.955	37416.07	0.002	0.999
6	0.923	37547.14	0.732	3.45×10^{-29}

© 2022 Bryan CJ et al. *JAMA Network Open*.

eTable 2. SITBI-R item response probabilities across 5 latent classes

	Class 1	Class 2	Class 3	Class 4	Class 5
**SITBI-R Items**	(n=8300)	(n=276)	(n=368)	(n=152)	(n=57)
Passive suicidal ideation					
I wish I could disappear or not exist	1.1	60.3	25.2	75.5	92.8
I wish I was never born	0.3	26.3	16.9	44.1	82.6
My life is not worth living	0.4	30.1	12.8	61.8	86.5
I wish I could go to sleep and never wake up	0.8	57.1	14.1	69.5	95.6
I wish I were dead	0.1	18.9	11.0	72.0	98.2
Active suicidal ideation					
Thoughts of killing yourself	0.3	22.2	30.3	84.3	96.3
Maybe I should kill myself	0.0	6.7	13.3	65.3	91.1
I should kill myself	0.0	0.0	11.1	37.5	96.1
I am going to kill myself	0.0	1.1	3.8	16.8	83.7
Suicidal planning					
Specific way or method	0.0	4.9	34.6	61.4	96
Specific place	0.0	1.4	12.3	24.1	88.6
Specific time	0.1	1.6	9.2	13.7	73.4
Suicidal behaviors					
Attempt to kill self with some intent to die	0.1	0.4	19.8	5.0	25.7
Preparatory behavior	0.1	0.3	44.4	12.1	56.2
Practice/rehearsal behavior	0.1	0.2	11.1	1.7	26.6
Aborted attempt	0.0	1.3	14.5	4.4	33.7
Interrupted attempt	0.0	0.2	8.6	3.9	29.0
Suicide attempt -- started then changed mind	0.0	0.4	7.8	0.0	28.4
Suicide attempt -- started then reached out for help	0.0	0.4	6.4	0.0	14.2
Suicide attempt -- found afterwards	0.1	0.0	6.4	0.0	16.0
Suicide attempt -- not found afterwards	0.0	0.4	5.5	0.8	19.5
Nonsuicidal self-injury					
Hurting self without wanting to die	0.4	6.2	9.4	15.8	36.0

Note: Darker shading reflects increasing probability of item endorsement.

© 2022 Bryan CJ et al. *JAMA Network Open*.
